# Ectodomain Architecture Affects Sequence and Functional Evolution of Vertebrate Toll-like Receptors

**DOI:** 10.1038/srep26705

**Published:** 2016-05-24

**Authors:** Jinlan Wang, Zheng Zhang, Jing Liu, Jing Zhao, Deling Yin

**Affiliations:** 1 Institute of Developmental Biology, School of Life Science, Shandong University, Jinan 250100, China; 2 State Key Laboratory of Microbial Technology, School of Life Science, Shandong University, Jinan 250100, China; 3 School of Pharmacy, Central South University, Changsha 410013, China; 4 Department of Internal Medicine, College of Medicine, East Tennessee State University, Johnson City, TN 37614, USA

## Abstract

Toll-like receptors (TLRs) are crucial components of innate immunity that specifically recognize diverse pathogen-associated molecular patterns from pathogens. The continuous hydrogen-bond network (asparagine ladder) formed among the asparagine residues on the concave surfaces of neighboring leucine-rich repeat modules assists in stabilizing the overall shape of TLR ectodomains responsible for ligand recognition. Analysis of 28 types of vertebrate TLRs showed that their ectodomains possessed three types of architectures: a single-domain architecture with an intact asparagine ladder, a three-domain architecture with the ladder interrupted in the middle, and a trans-three-domain architecture with the ladder broken in both termini. Based on a phylogenetic analysis, the three vertebrate TLR architectures arose during early evolution. The 1428 vertebrate TLRs can be divided into eight families based on sequence and structural differences. TLRs ligand specificities are affected by their ectodomain architectures. Three-domain TLRs bind hydrophobic ligands, whereas single-domain and trans-three-domain TLRs mainly recognize hydrophilic ligands. Analysis of 39 vertebrate genomes suggested that the number of single-domain TLR genes in terrestrial vertebrate genomes decreased by half compared to aquatic vertebrate genomes. Single-domain TLR genes underwent stronger purifying selective pressures than three-domain TLR genes in mammals. Overall, ectodomain architecture influences the sequence and functional evolution of vertebrate TLRs.

Gene duplications are believed to the primary driving force of evolutionary innovation. Functional divergence between duplicates is usually required for their long-term retention in the genome. The retained duplicates have three distinct fates: subfunctionalization, neofunctionalization, and subneofunctionalization^1^,^2^. However, the physical mechanisms producing functional divergences of duplicates are still less concerned.

Toll-like receptors (TLRs) are important pattern recognition receptors that play a crucial role in innate immunity in vertebrates^3^. TLR genes are present on the form of multi-copies in vertebrate genomes, for example, human has 10 TLRs and mouse has 12 TLRs. TLRs specifically recognize and bind a variety of highly conserved pathogen-associated molecular patterns (PAMPs) that are essential for pathogen survival, subsequently triggering protective immune responses against pathogen infections^4^,^5^. Numerous studies on human immunity have indicated that TLR genetic variation correlates closely with disease^6^,^7^. Functional studies have found that TLRs detect very diverse PAMP structures, including lipids, lipoproteins, proteins, and nucleic acids^8^,^9^. The high degree of evolutionary conservation of TLRs in vertebrates has enabled their phylogenetic clustering into receptor groups that are predicted to respond to similar ligands^10^.

TLRs are type I transmembrane glycoproteins that are expressed on the cell surface or in intracellular compartments. They generally consist of an N-terminal extracellular ligand-binding domain, a single transmembrane helix and a C-terminal intracellular toll–interleukin 1 receptor (TIR) domain that mediates signaling^11^. The TLR ectodomains (ECD) possess characteristic horseshoe-shaped solenoid structures generated by a varying number of leucine-rich repeat (LRR) modules. To date, several crystal structures of TLR ectodomains in complex with agonistic ligands have been determined (TLR1-TLR2-lipopeptide, TLR2-TLR6-lipopeptide, TLR3-dsRNA, TLR4-MD-2-LPS, TLR5-flagellin, TLR8-ssRNA, TLR9-CpG DNA, and TLR13-ssRNA)^12^,^13^,^14^,^15^,^16^,^17^,^18^,^19^. These structures emphasize the diversity and similarity of the interactions between paralogous TLRs and their ligands (Supplementary Fig. S1).

In the present study, we investigated the architectures of the vertebrate TLR ectodomains that are responsible for recognizing ligands. Based on comparative analyses of a large amount of sequence and structural information, we tried to reveal structural divergences, phylogenetic relationships, ligand preferences, and distribution characteristics in the vertebrate genomes among the different types of ectodomain architectures to provide a full-scale perspective of TLR evolution among the three levels of sequence, structure, and function.

## Results

### TLR ectodomains in vertebrates possess three types of architectures

**Figure 1:**
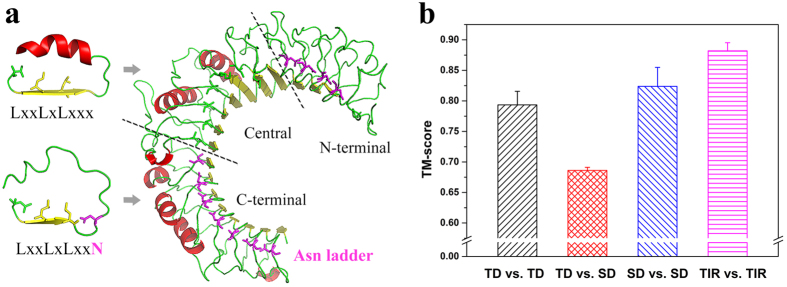
Significant structural differences in the ectodomains between single-domain TLRs and three-domain TLRs. (**a**) TLR ectodomains consist of numerous LRR modules that form a horseshoe shape. The asparagine ladder is colored in purple. For three-domain TLR2 (2Z7Xa), the asparagines in the conserved concave surface “LxxLxLxxN/CxL” motifs of the LRR modules are substituted in the central subdomain. “x” represents any amino acid. (**b**) Structural differences among TLR crystal structures. TM-scores in the columns represent the average degrees of structural differences of three-domain vs. three-domain TLR-ECDs (black), three-domain vs. single-domain TLR-ECDs (red), single-domain vs. single-domain TLR-ECDs (blue) and intracellular TIR vs. TIR domains (purple). Error bars represent SEMs.

The TLR ectodomains responsible for recognizing PAMPs possess more sequence and structural variability than the intracellular signaling domains. The typical LRR modules of ectodomains include 24 amino acid residues in which the conserved “LxxLxLxxN/CxL” motifs constitute the concave surface of horseshoe-shaped ectodomain (Fig. 1a). The conserved asparagines (Asn) in the concave surface are important for maintaining the shape of the entire ectodomain because they form a continuous hydrogen-bond network with backbone carbonyl oxygens of neighboring LRR modules; thus, they are visually defined as an asparagine ladder^20^,^21^. In a few cases, the asparagines can be substituted by other amino acids capable of donating hydrogens, such as threonine, serine, and cysteine.

Analysis of the known TLR-ECD crystal structures showed that the ectodomains of TLR3/5/8/9/13 possessed complete asparagine ladders; therefore, they were designated as possessing single-domain architecture (SD TLRs). However, the residues in the asparagine ladder positions in the middle of the ectodomains of TLR1/2/4/6 were substituted with other amino acids, indicating that the continuous hydrogen-bond networks were broken in the middle. Therefore, their ectodomains were divided into three distinctive regions: N-terminal and C-terminal subdomains with the asparagine ladder and a central subdomain lacking the asparagine ladder. Hence, the ectodomains of TLR1/2/4/6 were designated as having a three-domain architecture (TD TLRs, Fig. 1a). The central subdomains of three-domain TLRs possess atypical LRR modules that generally lack phenylalanine spines. Moreover, these LRR modules vary considerably in length and possibly form one or more helices that are inserted into the convex area of the longer LRR modules^22^,^23^.

Pairwise comparisons of TLR crystal structures were performed with TM-align (Fig. 1b). The average TM-score of pairwise comparisons among TIR domains was nearly 0.9, indicating that the known TIR domains were very similar. This finding is expected because the functions of intracellular TIR domains are conserved. The structural differences among ectodomains were larger than those among TIR domains, but the TM-score of random pairwise ectodomains was higher than 0.5, indicating that they assume the same fold. The TM-scores of pairwise comparisons between three-domain and single-domain TLRs were significantly lower than those within the same TLR architecture, suggesting that the conformations of three-domain TLR ectodomains deviated strongly from those of single-domain TLR ectodomains. The ectodomains of three-domain TLRs had smaller radii than those of single-domain TLRs, and the structural distortions of their central subdomains formed hydrophobic internal pockets at the borders between the central and C-terminal subdomains (Supplementary Fig. S2).

Next, the module compositions of the representative sequences of 28 types of primary TLR ectodomains in vertebrates were thoroughly analyzed. For those TLRs with unknown crystal structures, we modeled their structures using the threading approach (Supplementary Table S1). The results of the LRR prediction showed that vertebrate TLR ectodomains generally consisted of 12–25 LRR modules that were flanked by LRRNT and LRRCT modules at both termini (Supplementary Table S2). TLR1LB included only 12 LRRs, TLR1/1LA/2/2A/2B/6/10/14/15/18/25 included 19 LRRs, TLR4/5/5S/5SL included 21 LRRs, TLR3/11/12 included 23 LRRs, TLR19/20 included 24 LRRs, and TLR7/8/9/13/21/22/23 included 25 LRRs.

**Figure 2:**
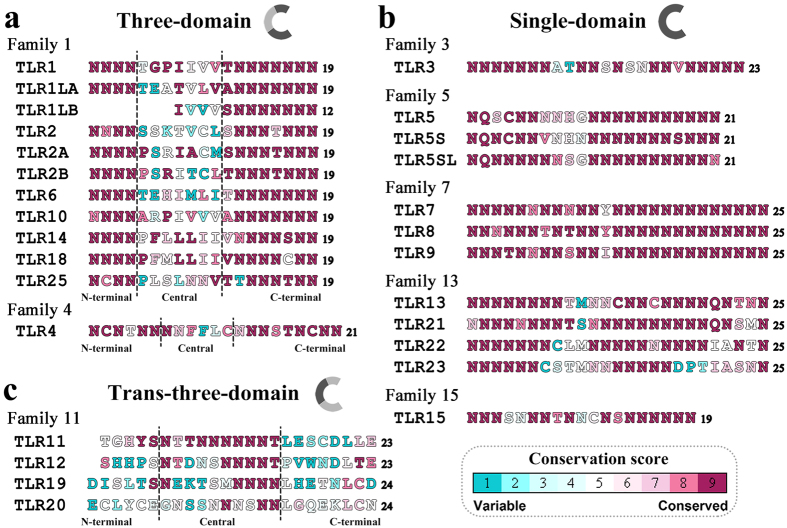
Three composition patterns of the asparagine ladders in TLR ectodomains. (**a**) Twelve TLR-ECDs possess a three-domain architecture. The residues in the asparagine ladder positions of their LRR modules were highly conserved asparagines in the N-terminal and C-terminal subdomains, but they were substituted with other variable amino acid residues in the central subdomains. (**b**) Twelve TLR-ECDs possess a single-domain architecture. The residues in the asparagine ladder positions of their LRR modules throughout the entire ectodomain were almost always highly conserved asparagines. (**c**) Four TLR-ECDs possess a trans-three-domain architecture. The residues in the asparagine ladder positions of their LRR modules were substituted with other variable amino acid residues in the N-terminal and C-terminal subdomains, but most of them were relatively conserved asparagines in the central subdomains. Vertebrate TLRs with the same number are generally orthologous. For each TLR ectodomain, the highest frequency amino acid residue that occurs in the asparagine ladder positions of each LRR module was successively displayed on the basis of multiple sequence alignments among homologous sequences. Different colors represent the estimated evolutionary conservation of each ladder position. The conservation scale is defined from the most variable positions (grade 1, colored turquoise) to the most conserved positions (grade 9, colored maroon). The number of LRR modules in the ectodomains is listed in the last column.

The compositions of the asparagine ladders in these TLR ectodomains were subsequently determined based on module predictions (Fig. 2). The degree of evolutionary conservation of each residue position in the asparagine ladder was estimated. The results showed that most of the ladder positions consisted of highly conservative asparagines, whereas a few ladder positions consisted of other variable amino acids. Similar to the three-domain TLR1/2/4/6 with reported crystal structures, the asparagine ladders of TLR1LA/2A/2B/10/14/18/25 were also interrupted in the middle, suggesting that their ectodomains also possibly belonged to the three-domain architecture group (Fig. 2a). Avian TLR1LB is unique and lacks the first seven LRR modules compared to TLR1LA. Thus, it most likely represents a variant of the three-domain architecture. In contrast, the asparagine ladders throughout the ectodomains of TLR5S/5SL/7/15/21/22/23 were relatively intact, which were similar to the cases of single-domain TLR3/5/8/9/13 with known crystal structures. Thus, we speculated that their ectodomains also possessed single-domain architecture (Fig. 2b). The phenylalanine spines of the single-domain TLR ectodomains were generally complete, whereas the phenylalanine spines in the atypical LRR modules in the central subdomain were lacking in three-domain TLR ectodomains (Supplementary Fig. S3). There were also significant structural differences of the predicted structures between the three-domain TLR-ECDs and the single-domain TLR-ECDs (Supplementary Fig. S2).

Interestingly, the asparagine ladders throughout the TLR11/12/19/20 ectodomains were broken in both the C-terminal and N-terminal subdomains, but they were intact in the central subdomain (Fig. 2c). This was markedly different from those of the known single-domain and three-domain architectures, and likely signifies a new, unknown type of ectodomain architecture. Because the ectodomains of TLR11/12/19/20 could be split into three sections and the compositions of their asparagine ladders were opposite to those of the known three-domain architectures, they were defined as having trans-three-domain architectures (TTD TLRs). Hence, the ectodomains of vertebrate TLRs can be assigned to at least three types of architectures based on sequence and structural differences.

### The three types of vertebrate TLR architectures diverged during early evolution

**Figure 3:**
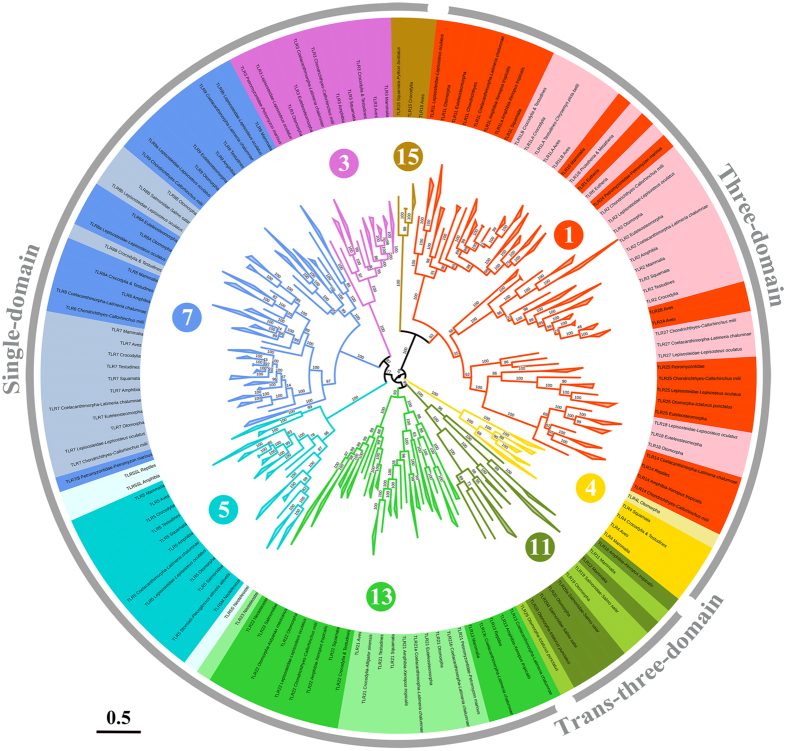
Phylogenetic relationships among vertebrate TLRs. The phylogenetic tree was built using 1428 vertebrate TLR amino acid sequences, and its robustness was estimated by the aLRT SH-like method. Branches of each major family are shown in a unique color. The architecture of each family and the TLRs included in each family are also labeled. To avoid crowding, some branches are collapsed. The tree is drawn to scale, with branch lengths in the same units as those of the evolutionary distances used to infer the phylogenetic tree.

Almost all known vertebrate TLR sequences were acquired from the database (Supplementary Data S1). These 1428 TLR sequences from 221 species covered all of the major vertebrate taxa. The phylogenetic relationships of these vertebrate TLR sequences were determined using the PhyML approach (Fig. 3). A preliminary study showed that nearly all vertebrate TLRs were included into six major families^10^. Here, the phylogenetic relationships constructed using ~15 times more TLR sequences than used in the preliminary study were similar to the relationships identified in the latter. However, we propose the further division of vertebrate TLRs into eight families based on possible structural differences among TLR ectodomain architectures: families 1/3/4/5/7/11/13/15 (defined by the lowest ordinal TLR contained in that family).

Family 1 includes TLR1/1LA/1LB/2/2A/2B/6/10/14/18/24/25 as well as TLR27^24^. These TLR ectodomains possess the three-domain architecture. In addition to TLR1LB, the ectodomains of the other TLRs in family 1 contain 19 LRRs. Although the TLR15 ectodomain also possessed 19 LRRs, it was distinct from the other members of family 1 in the evolutionary tree. Also, because its asparagine ladder was intact, the ectodomain of TLR15 is considered to be more similar to the single-domain architecture. Therefore, TLR15 should be considered as an individual family (family 15) based on structural differences.

Family 3 includes only TLR3, whose ectodomain possesses the single-domain architecture and contains 23 LRRs. Family 4 contains only TLR4, whose ectodomain possesses the three-domain architecture and 21 LRRs. Family 5 includes TLR5/5S/5SL. Although TLR5S/5SL lack transmembrane and intracellular domains, both of their ectodomains possess the single-domain architecture and include 21 LRRs (similar to TLR5). Family 7 includes TLR7/8/9, the ectodomains of which also possess the single-domain architecture and 25 LRRs.

Family 11 is unique because TLR11/12/16/19/20/26 and TLR13/21/22/23 are divided into two clades at the root of the phylogenetic tree. The asparagine ladders of the ectodomains of TLR11/12/16/19/20/26 are broken in the C-terminal and N-terminal subdomains. Thus, their ectodomains may possess a trans-three-domain architecture. In contrast, the ectodomains of TLR13/21/22/23 possess intact asparagine ladders. Thus, they may possess the single-domain architecture. Hence, we propose that the original family 11 is split into two families: the new family 11, including TLR11/12/16/19/20/26; and family 13, including TLR13/21/22/23.

Phylogenetic analysis showed that the ectodomains of TLRs belonging to the same family nearly always contained the same number of LRR modules and possessed the same architecture (Fig. 2). Furthermore, families 3/5/7/13, in which the TLR ectodomains possessed the single-domain architecture, neighbored with each other in the tree. Moreover, families 1/4, in which the TLR ectodomains possessed the three-domain architecture, were also neighbors in the tree. Therefore, in addition to family 15, three types of vertebrate TLR ectodomain architectures may have separated during early evolution.

### Ectodomain architecture affects TLR ligand-binding specificities

The ectodomains of vertebrate TLRs mediate the recognition of PAMPs. Although only the ligand-binding specificities of partial TLRs have been studied to date and although there may be some overlap in the ligands recognized by different TLRs, different ectodomain architectures evidently affect the ligand-binding specificities of TLRs (Supplementary Table S3).

TD family 1 is the biggest TLR family and includes approximately one-third of the total number of known vertebrate TLRs. Family 1 can be further divided into three groups based on phylogenetic relationships (Fig. 3). Group A includes TLR1/6/10, group B contains TLR2/24, and group C encompasses TLR14/18/25/27. At least one representative from group A and group B has been found in all vertebrate taxa. Studies to date have shown that all known TLRs from group A are able to form functional heterodimers with the known TLRs from group B on the cell surface, and together these TLRs recognize hydrophobic molecules such as lipids and lipoproteins^25^,^26^,^27^. However, TLRs from group C have not been found in Mammalia and Aves, and their functional roles are still unclear.

TLR3 in SD family 3 exists in all vertebrate taxa as a single gene copy in each species’ genome. Hence, family 3 is the most conservative TLR family. TLR3 responds to double-stranded RNA (dsRNA) by forming homodimers in intracellular vesicles^28^.

TLR4 in TD family 4 has been reported in Reptilia, Mammalia and Aves. TLR4 homodimers recognize lipopolysaccharides (LPS) on the cell surface with the help of two MD-2 coreceptors^29^. However, some teleosts possess multiple copies of TLR4L, which is similar to TLR4 but does not recognize LPS^30^.

TLR5 in SD family 5 is expressed on the cell surface and recognizes bacterial flagellin in the form of a homodimer^31^. In addition to TLR5, a type of soluble TLR5 (TLR5S) has been found in Neoteleostei. TLR5S is a paralogue of TLR5(M) and is also able to recognize bacterial flagellin^32^. Furthermore, some amphibians and reptiles possess a type of TLR5SL that is analogous to the fish TLR5S, but phylogenetically distant from TLR5/5S.

TLR7 in SD family 7 has been identified throughout the major vertebrate taxa, whereas both the TLR8 and TLR9 genes are lost from the avian genomes. Both TLR7 and TLR8 mediate the recognition of single-stranded RNA (ssRNA); moreover, TLR8 homodimers were recently verified as being able to sense ssRNA degradation products^17^,^33^,^34^. TLR9 homodimers recognize agonistic unmethylated CpG-containing DNA^35^.

The known members of TTD family 11 only exist in a few vertebrate taxa. Both TLR11 and TLR12 are only found in mammals, whereas TLR16 is unique to *Xenopus* and TLR19, TLR20 and TLR26 have only been found in some species of teleost^36^. Mouse TLR11 and TLR12 colocalize to similar endosomal compartments and are able to form homo- and hetero-dimers to recognize profilin from *Toxoplasma gondii*^37^,^38^. The gene expressions of TLR19 and TLR20 were significantly induced by the protozoan parasitic infection^39^,^40^.

SD family 13 can be divided into two groups based on phylogenetic relationships: group A including TLR13/21 and group B including TLR22/23 (Fig. 3). Intriguingly, the ligands recognized by group A and group B partially overlap with the ligands recognized by family 7 and family 3, respectively. For instance, TLR13 is able to respond to ssRNA^41^, similar to TLR7/8, whereas TLR21 recognizes unmethylated CpG-containing DNA, similar to TLR9^42^. TLR22 also recognizes dsRNA, similar to TLR3^43^.

TLR15 is the sole member in family 15 and is unique to avian and partial reptilian lineages. TLR15 recognizes secreted virulence-associated fungal and bacterial proteases^44^. However, in contrast to other known TLR-activated mechanisms, the cleavage of the TLR15 ectodomain by microbial proteases has been proposed to result in the release of inhibitory elements, thereby causing TLR15 self-activation without the requirement for an external ligand.

Taken together, three-domain TLRs were found to mainly recognize hydrophobic ligands, the majority of which are microbial membrane components such as lipids, lipoproteins, and LPS. In contrast, single-domain and trans-three-domain TLRs chiefly respond to hydrophilic ligands, such as nucleic acids and proteins. Three-domain TLRs are mainly expressed on the cell surface, whereas single-domain and trans-three-domain TLRs are chiefly expressed in intracellular vesicles, such as endosomes. Furthermore, both three-domain and trans-three-domain TLRs have been reported to possess some TLRs that work in the forms of functional heterodimers, whereas all known single-domain TLRs have been reported to form homodimers to bind ligands.

### The three types of TLR architectures may possess different functional importance in vertebrate taxa

**Figure 4:**
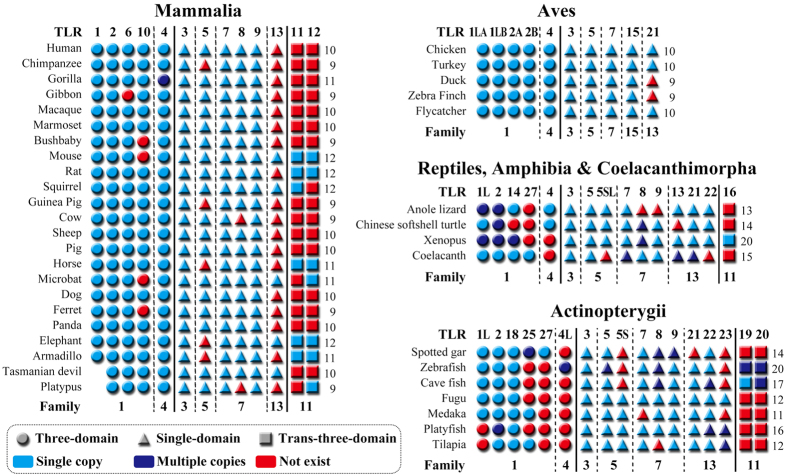
TLR genes in representative vertebrate species with a sequenced genome. TLR genes in 23 mammalian genomes, 5 avian genomes, 2 reptilian genomes, 1 amphibian genome, 1 Coelacanthimorpha genome, and 7 Actinopterygii genomes were analyzed. The circles represent three-domain TLRs, the triangles signify single-domain TLRs, and the squares represent trans-three-domain TLRs. The single copy and multiple copy genes in the genomes are colored light blue and dark blue, respectively. Red represents pseudogenes or genes that do not exist in each genome. The total number of TLR functional genes in the genomes for each species is listed in the far-right column. TLR family information is also given. The early gene duplication from which the TLR1 and TLR6 genes originated did not occur in the platypus and Tasmanian devil.

The distribution of TLR genes was analyzed in 39 vertebrate genomes (Supplementary Table S4). The results showed that an individual vertebrate genome may possess 9~20 functional TLR genes and that there may be large differences in the number and types of TLR genes among different vertebrate genomes (Fig. 4). The genes encoding three-domain and single-domain TLRs occupy the vast majority of TLR genes in vertebrate genomes; in contrast, trans-three-domain TLR genes only occur in a few vertebrate genomes. We found that the number of single-domain TLR genes was approximately twice the number of three-domain TLR genes in fish (Actinopterygii) genomes, whereas the numbers were approximately equal in both mammalian and avian genomes. The reason for this difference may be that the number of single-domain TLR genes is reduced in terrestrial vertebrate genomes. For example, the average number of single-domain TLR genes was 9.6 in fish genomes and 4.9 in mammalian genomes. This finding suggests that fish require more single-domain TLRs to respond to pathogenic microorganisms in aquatic environments.

**Figure 5:**
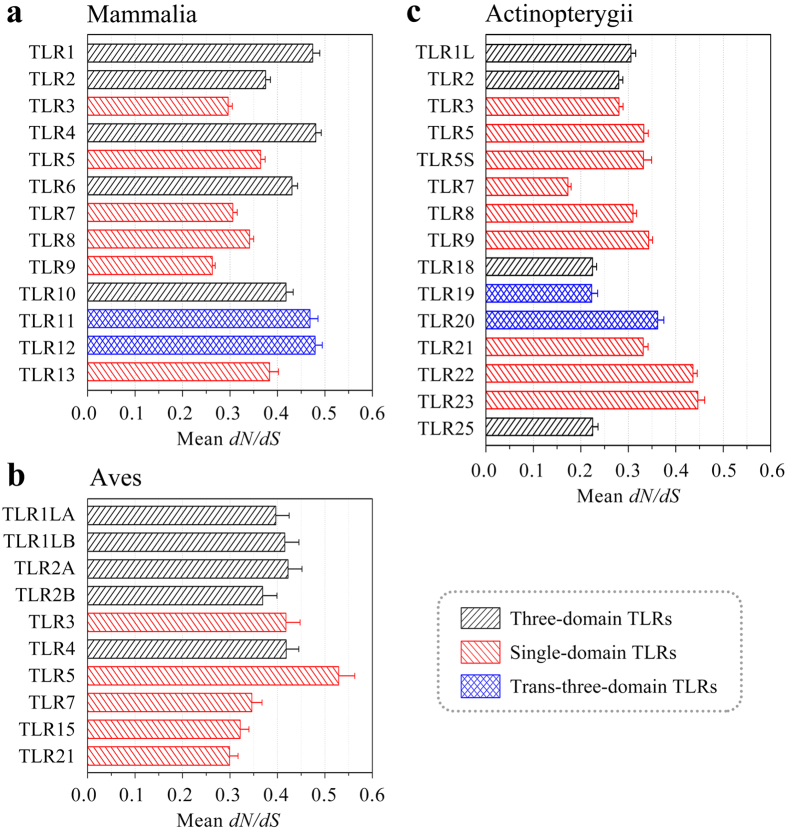
Mean *dN/dS* values of TLR loci in the genomes of Mammalia (**a**), Aves (**b**) and Actinopterygii (**c**). Three-domain TLRs, single-domain TLRs, and trans-three-domain TLRs are represented in black, red, and blue, respectively. Error bars represent 95% confidence intervals for the mean *dN/dS* values.

The average *dN/dS* value at each TLR locus in the genomes of Mammalia, Aves and Actinopterygii was estimated (Fig. 5). The *dN/dS* value is the ratio of the number of nonsynonymous substitutions per nonsynonymous site (*dN*) to the number of synonymous substitutions per synonymous site (*dS*), it can assess the functional constraint differences acting on distinct TLR genes. The results showed that the mean *dN*/*dS* values at TLR loci in the three taxa ranged from 0.173 to 0.529, suggesting that the evolution of vertebrate TLR genes was mainly dominated by purifying selection and a low proportion of non-synonymous substitutions helped preserve the biological function of the TLRs. The *dN*/*dS* values of single-domain TLR loci were significantly lower than those of the three-domain and trans-three-domain TLR loci in mammalian genomes, suggesting that single-domain TLRs may have been subjected to stronger functional constraints (Fig. 5a). There appeared to be no significant differences in the *dN*/*dS* values between single-domain TLR loci and three-domain TLR loci in the avian genomes (Fig. 5b). However, in fish genomes the *dN*/*dS* values of TLR loci sharing the same architecture varied considerably. Although the *dN*/*dS* value was the lowest for the TLR7 locus, overall, the functional constraints acting on three-domain TLR loci were slightly stronger than those acting on single-domain TLR loci (Fig. 5c). In summary, TLRs with the three types of architectures possess different functional importance in vertebrate taxa.

## Discussion

The early concept of innate immunity suggested that it nonspecifically recognized microbes; however, the discovery of TLRs showed that pathogen recognition by the innate immune system was actually specific^7^. The conserved asparagines in the concave surfaces of LRR modules are crucial for maintaining the overall shape of the TLR ectodomains that are responsible for ligand recognition^20^,^22^,^23^. The ectodomains of single-domain TLRs with complete asparagine ladders occur approximately in a plane, and thus are probably suitable for binding hydrophilic nucleic acids or proteins. Three-domain TLRs with the broken asparagine ladders in the middle of their ectodomains are more suitable for binding hydrophobic ligands because the structural transitions of the three-domain TLR ectodomains at the boundary between the central and the C-terminal subdomains expose a large hydrophobic pocket. Several crystal structures of TLRs with agonists reflected these features^12^,^13^,^14^,^15^,^16^,^17^,^18^,^19^. Despite the lack of crystal structures, functional studies have suggested that trans-three-domain TLRs with broken asparagine ladders in both ectodomain termini parts may chiefly recognize protein ligands^37^,^38^.

A large number of vertebrate TLRs that have not yet been named nor have clear classifications were identified based on our full-scale phylogenetic analysis (Supplementary Data S1). We proposed that the known vertebrate TLRs can be categorized into eight families according to sequence and structural differences. TLR ectodomains in the same family possess the same architecture and recognize similar ligands. In addition, we found that the three types of ectodomain architectures have separated during early evolution. These ancient architectures appear to have specific corresponding relationships with different groups of pathogenic microorganisms. Three-domain TLRs mainly recognize PAMPs from bacteria and fungi, whereas the majority of ligands for single-domain TLRs are derived from viruses. Trans-three-domain TLRs chiefly respond to ligands from protozoan parasites. However, the members of family 5 with the single-domain architecture are exceptions, because they recognize bacterial flagellin^31^,^32^. TLR15 is even more unique, because it neighbors with three-domain TLRs in the phylogenetic tree, but its ectodomain resembles the single-domain architecture. It is noteworthy that the unique activation mechanism of TLR15 through the cleavage of its ectodomain by microbial proteases, requiring no extra ligand, differs from other TLRs^44^.

Vertebrates underwent the transition from aquatic to terrestrial environments during the evolutionary process. Compared to terrestrial vertebrates, fish depends mainly on the innate immune response in combating high concentrations of pathogens in the aquatic environment due to an undeveloped adaptive immune response^45^,^46^. We found that the number of TLR genes was larger in fish genomes than in terrestrial vertebrate genomes (i.e., birds, reptiles and mammals). Compared to fish species, the number of single-domain TLR genes in terrestrial vertebrate genomes has decreased by half. Furthermore, the *dN*/*dS* values revealed that the evolution of vertebrate TLRs was chiefly dominated by purifying selection. This is consistent with the function that TLRs recognize the conserved molecular motifs in pathogens. The purifying selection pressures acting on single-domain TLR loci were much stronger than those acting on the loci of the other two types of TLR architectures in mammals, whereas the opposite case appears to have occurred in fish. Based on structural similarities, the ligands recognized by TLRs with the same architecture may partly overlap with one another. The existence of multiple single-domain TLR loci may mutually decrease the functional constraints acting on each single-domain TLR locus in fish genomes.

Overall, we provided a full-scale perspective of vertebrate TLR evolution among the three levels of sequence-structure-function. Three different types of vertebrate TLR architectures emerged during early evolution. These structural distinctions in the ectodomains may have provided the basis for the functional divergences for TLRs that recognize different PAMPs.

## Materials and Methods

### TLR information acquisition

The coding sequences of vertebrate TLR genes were acquired from the NCBI (GenBank) and Ensembl databases. All partial sequences (<1800 bp) and pseudogene sequences were excluded. The taxonomic designations of species from which each TLR gene sequence originated were obtained from the NCBI Taxonomy database. In total, 1428 TLR sequences from 221 vertebrate species were collected (Supplementary Data S1). The distribution of TLR genes in the genomes was analyzed using 39 vertebrate species with sequenced genomes. All TLR gene information in these genomes was confirmed by means of mutual calibration between the NCBI and Ensembl databases (Supplementary Table S4). The crystal structures of TLRs were obtained from the PDB database.

### Modeling TLR structures

I-TASSER was used to predict the structures of ectodomains and TIR domains for TLRs that had unsolved crystal structures (Supplementary Table S1)^47^. I-TASSER is a hierarchical method for protein structure prediction. Structural templates were first identified from the PDB by the multiple-threading program LOMETS; then, full-length models were constructed by iterative template fragment assembly simulations.

### Structural comparison

Pairwise structural differences of TLRs were measured with TM-align^48^. The following reported TLR crystal structures were used for automated structural alignment: ectodomains, 2Z7Xb, 2Z7Xa, 3A79a, 2A0Za, 3CIYa, 3FXIa, 3VQ2a, 3A79b, 3W3Ka, 3WPFa, 3WPEa, 3WPCa, and 4Z0Ca; intracellular TIR domains, 1FYVa, 4OM7a, 2J67a, 1FYXa, and 3J0Aa. The TM-score was used to measure the structural similarity of two protein structures^49^ and had the value (0, 1). The higher the TM-score, the more similar the two aligned TLR structures. The TM-score was normalized by protein length; therefore, two scores are reported for each pairwise comparison. The average TM-score for each pairwise comparison was used for further statistical analysis.

### Identification of TLR structural elements

First, we identified the structural elements of the representative sequence for each type of TLR. The signal peptide, ectodomains, transmembrane region, and intracellular TIR domains were predicted by SignalP, SMART, and TMHMM^50^,^51^,^52^. The delimitation of each LRR module in the TLR ectodomains was defined by LRRfinder (Supplementary Table S2)^53^. The structural information for the TLR ectodomains was used for the calibration of LRR module prediction. The structural element delimitations of these representative TLR sequences were used as references for other orthologous TLRs of the remaining species. Then, the structural element compositions of other orthologous TLRs were identified through multiple sequence alignments.

### Phylogenetic analysis

A multiple sequence alignment of the full-length protein sequences of vertebrate TLRs was implemented using MAFFT (FFT-NS-i, BLOSUM62)^54^. The phylogenetic tree was constructed using PhyML with the LG substitution model and four substitution rate categories^55^. Estimations of branch supports were calculated with approximate likelihood ratio tests (aLRT SH-like)^56^. The phylogenetic tree was visualized by iTOL^57^.

### Evolutionary analysis

A multiple sequence alignment of the nucleic acid sequences of the TLR genes, based on their codons, was constructed by TranslatorX and MAFFT^58^. The ratio of the number of nonsynonymous substitutions per nonsynonymous site (*dN*) to the number of synonymous substitutions per synonymous site (*dS*), *dN*/*dS*, an indicator of the selective pressure acting on a protein-coding gene, at the TLR loci and the corresponding 95% confidence intervals were calculated with the Datamonkey web server^59^. The evolutionary conservation of the asparagine ladder positions in TLR proteins was estimated using the ConSurf algorithm^60^. The LG substitution matrix and the empirical Bayesian paradigm were used for the accuracy estimations of the conservation scores. The continuous conservation scores were divided into a discrete scale of nine grades for visualization, from the most variable positions (grade 1) to the most conserved positions (grade 9). The evolutionary analysis was not performed for TLR16/24/26/27, because the number of their known full-length sequences was less than 5.

## Additional Information

**How to cite this article**: Wang, J. *et al*. Ectodomain Architecture Affects Sequence and Functional Evolution of Vertebrate Toll-like Receptors. *Sci. Rep*. **6**, 26705; doi: 10.1038/srep26705 (2016).

## Supplementary Material

Supplementary Information

Supplementary Dataset 1
