# Supplementary material for: Ectodomain Architecture Affects Sequence and Functional Evolution of Vertebrate Toll-like Receptors
Source: Sci Rep. 2016 May 24;6:26705. doi: 10.1038/srep26705 (PMC4877655; doi:10.1038/srep26705)
Supplement: Supplementary Information [file srep26705-s1.pdf]

Supplementary Information

Ectodomain Architecture Affects Sequence and Functional Evolution of Vertebrate Toll-like Receptors

Jinlan Wang[1,*], Zheng Zhang[2,*], Jing Liu[1], Jing Zhao[1] & Deling Yin[3,4]

[1]Institute of Developmental Biology, School of Life Science, Shandong University, Jinan 250100, China. [2]State Key Laboratory of Microbial Technology, School of Life Science, Shandong University, Jinan 250100, China. [3]Department of Pharmacology, School of Pharmaceutical Sciences, Central South University, Changsha 410078, China. [4]Department of Internal Medicine, College of Medicine, East Tennessee State University, Johnson City, TN 37614, USA.

[*]These authors contributed equally to this work.

Correspondence and requests for materials should be addressed to Z.Z. (email: zhzhang.sdu@gmail.com) or D.Y. (email: yin@etsu.edu).

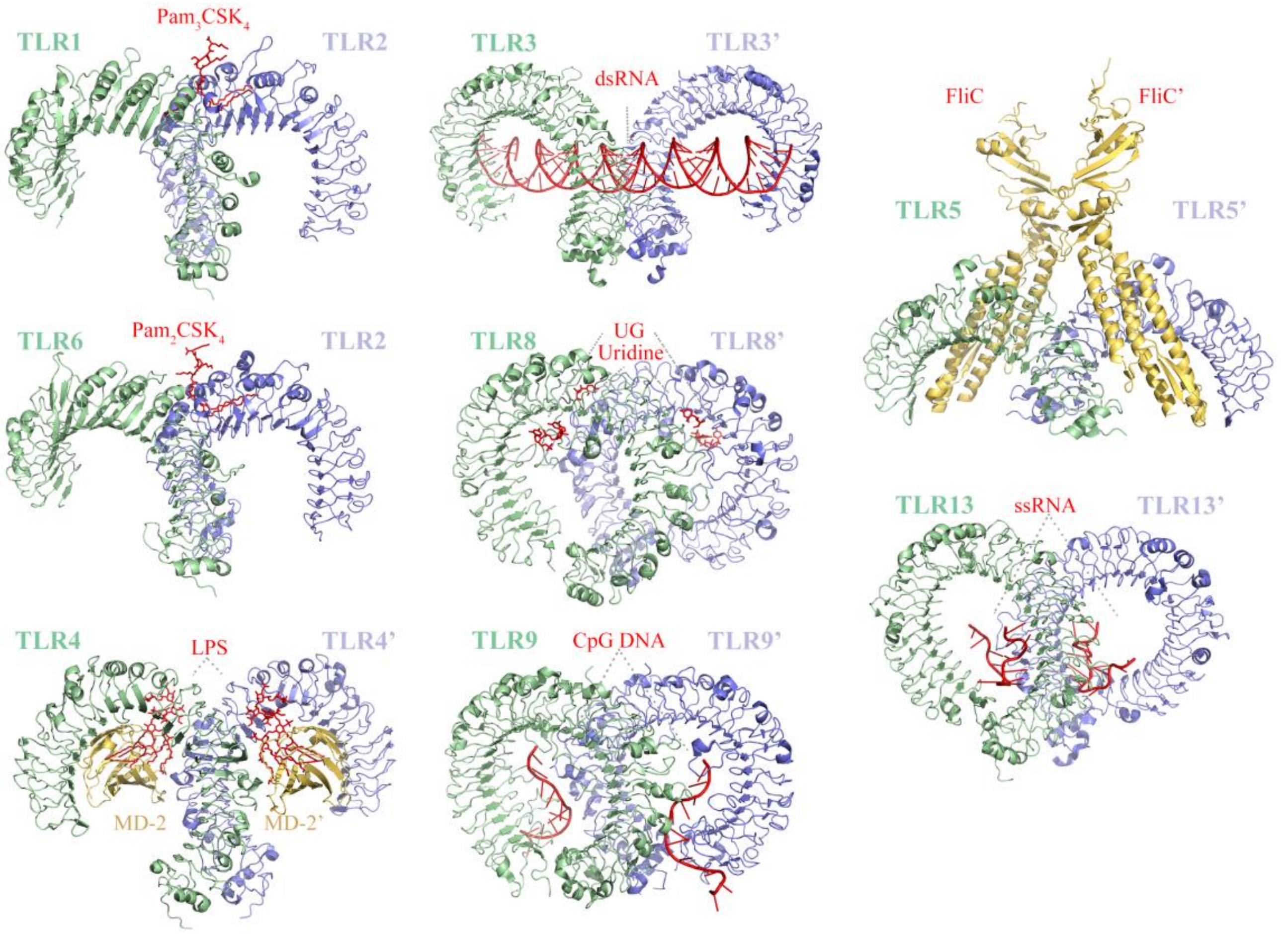

Supplementary Figure S1. Known crystal structures of TLRs in complex with agonists. Structural comparison of the ligand-bound dimers of TLR1-TLR2-lipopeptide (PDB code: 2Z7X), TLR2-TLR6-lipopeptide (PDB code: 3A79), TLR3-dsRNA (PDB code: 3CIY), TLR4-MD-2-LPS (PDB code: 3FXI), TLR5-flagellin (PDB code: 3V47), TLR8-ssRNA degradation products (PDB code: 4R08), TLR9-CpG DNA (PDB code: 3WPC), and TLR13-ssRNA (PDB code: 4Z0C). $Pam_2CSK_{4,}$ diacylated lipopeptide; $Pam_3CSK_{4,}$ triacylated lipopeptide; dsRNA, double-stranded RNA; LPS, lipopolysaccharide; Flic, flagellin; Uridine (1st site) and UG (2st site), the degradation products of single-stranded RNA; CpG DNA, unmethylated CpG-containing DNA; ssRNA, single-stranded RNA.

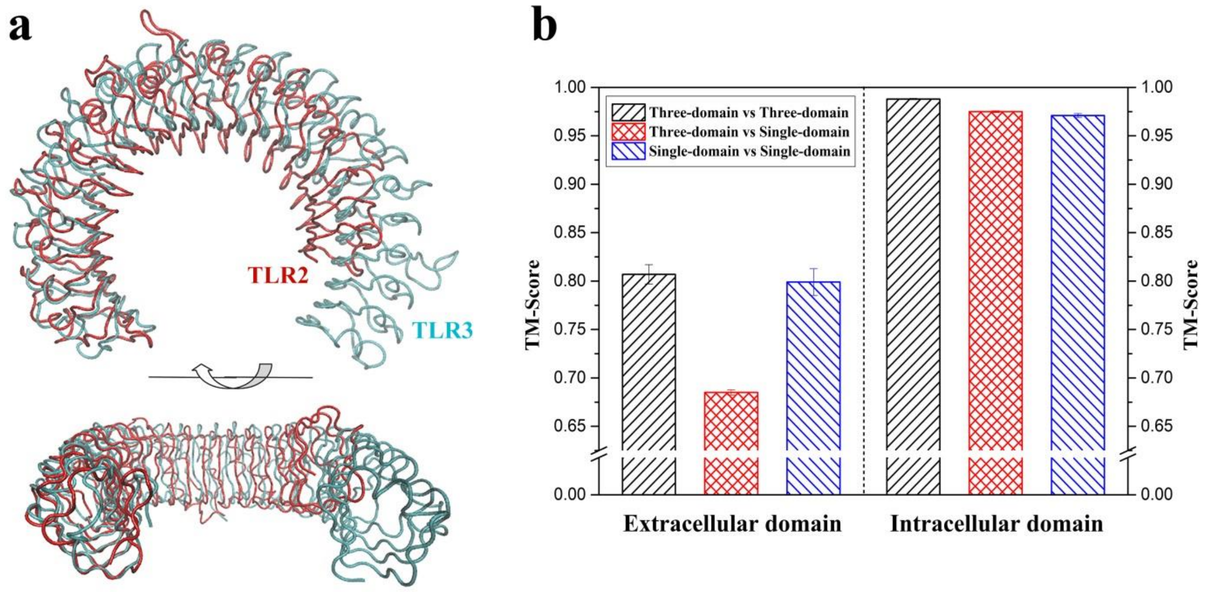

Supplementary Figure S2. Significant structural differences in the ectodomains between single-domain TLRs and three-domain TLRs. (**a**) Superposition of TLR ectodomains. Three-domain TLR2 (PDB code: 2Z7Xa, red) forms a smaller radius than single-domain TLR3 (PDB code: 3CIY, cyan) due to the structural distortions located in its central subdomain. Side view (upper panel) and top view (lower panel). (**b**) Average structural differences among the modeled TLR structures estimated by TM-align. The sequence information used for modeling is listed in Supplementary Table S1. TM-scores in the columns represent the average degrees of structural differences of three-domain vs. three-domain TLRs (black), three-domain vs. single-domain TLRs (red), and single-domain vs. single-domain TLRs (blue). The structural differences among ectodomains (left) and intracellular domains (right) are displayed. Error bars represent the SEMs.

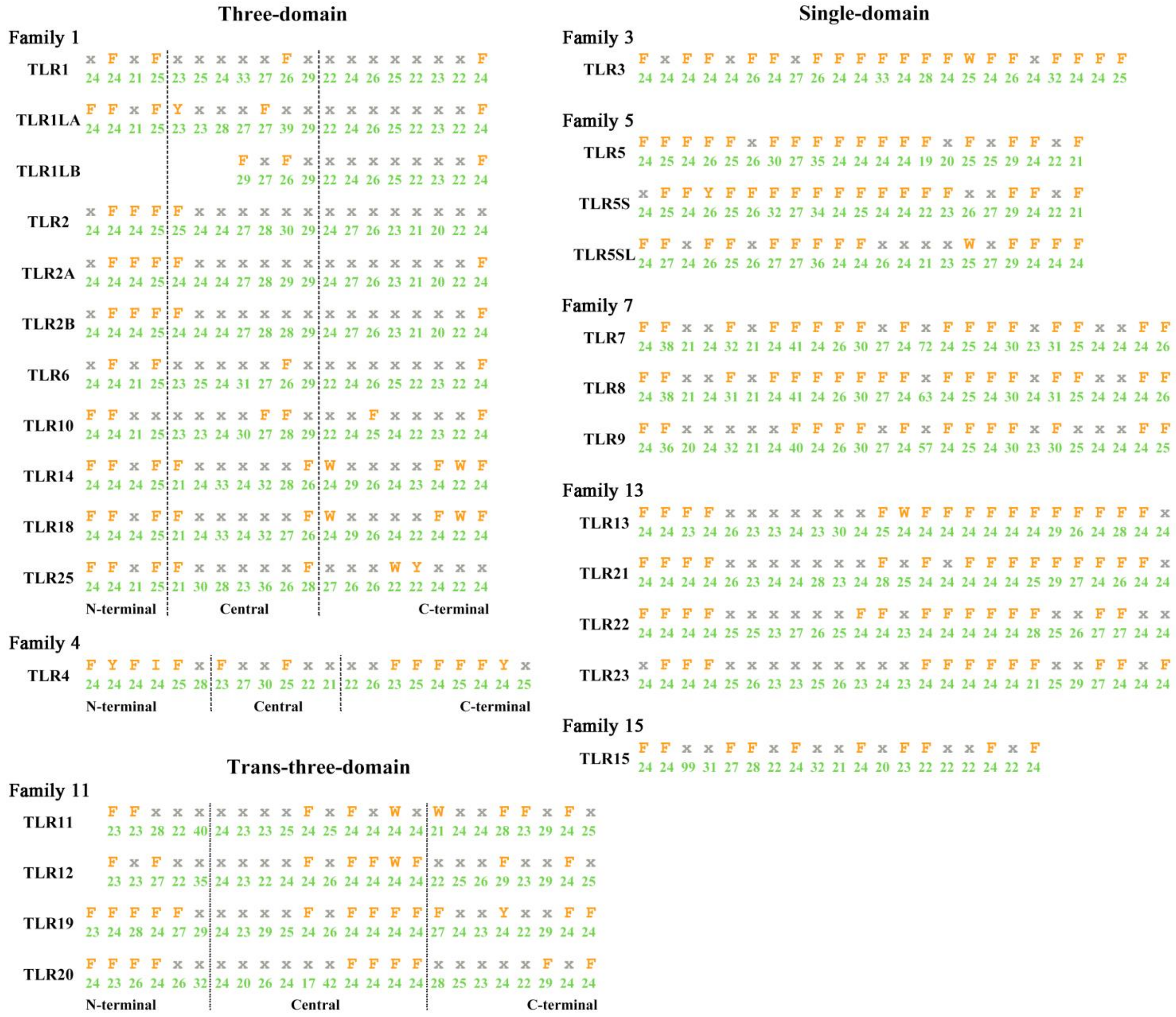

Supplementary Figure S3. Compositions of phenylalanine spines and the length of each LRR module within the TLR ectodomains. The ectodomains of 28 types of vertebrate TLRs were analyzed. The representative sequence information for 28 types of vertebrate TLRs and the results of their module predictions are listed in Supplementary Tables S1 and S2, respectively. For each TLR ectodomain, the highest frequency amino acid residue that occurs in the phenylalanine spine positions of each LRR module is successively displayed based on the multiple sequence alignments among homologous sequences. "x" represents any amino acid residue except aromatic amino acids, implying that the phenylalanine spine is lacking in the corresponding LRR module. The length of each LRR module within the ectodomains of representative sequences of 28 types of vertebrate TLRs is displayed together. The family and ectodomain architecture for each type of TLR are labeled. This analysis was not performed for TLR16/24/26/27 because the number of their known full-length sequences was less than 5.

Supplementary Table S1. Modeling of representative sequences of 28 types of vertebrate TLRs.

TLR	Scientific name	Accession No.	Modeled ectodomains	
			Length (aa)	Estimated TM-score*
TLR1	*Homo sapiens*	ENSG00000174125	557	0.66±0.13
TLR1LA	*Gallus gallus*	ENSGALG00000017485	565	0.65±0.13
TLR1LB	*Gallus gallus*	ENSGALG00000027093	411	0.61±0.14
TLR2	*Homo sapiens*	ENSG00000137462	567	0.88±0.07
TLR2A	*Gallus gallus*	XM_003641158	571	0.81±0.09
TLR2B	*Gallus gallus*	AB046533	562	0.85±0.08
TLR3	*Homo sapiens*	ENSG00000164342	680	0.90±0.06
TLR4	*Homo sapiens*	ENSG00000136869	610	0.99±0.04
TLR5	*Homo sapiens*	ENSG00000187554	623	0.91±0.06
TLR5S	*Takifugu rubripes*	XM_003971921	620	0.90±0.06
TLR5SL	*Xenopus tropicalis*	ENSXETG00000030446	635	0.71±0.12
TLR6	*Homo sapiens*	ENSG00000174130	555	0.67±0.13
TLR7	*Homo sapiens*	ENSG00000196664	816	0.78±0.09
TLR8	*Homo sapiens*	ENSG00000101916	799	0.92±0.06
TLR9	*Homo sapiens*	ENSG00000239732	793	0.77±0.10
TLR10	*Homo sapiens*	ENSG00000174123	557	0.66±0.13
TLR11	*Mus musculus*	ENSMUSG00000051969	688	0.66±0.13
TLR12	*Mus musculus*	ENSMUSG00000062545	688	0.73±0.11
TLR13	*Mus musculus*	ENSMUSG00000033777	715	0.92±0.06
TLR14	*Anolis carolinensis*	XM_003230485	575	0.69±0.12
TLR15	*Gallus gallus*	NM_001037835	631	0.72±0.11
TLR18	*Danio rerio*	ENSDARG00000040249	576	0.65±0.13
TLR19	*Ictalurus punctatus*	HQ677722	714	0.68±0.12
TLR20	*Ictalurus punctatus*	HQ677723	715	0.71±0.11
TLR21	*Gallus gallus*	ENSGALG00000000774	714	0.82±0.08
TLR22	*Danio rerio*	NM_001128675	720	0.78±0.10
TLR23	*Takifugu rubripes*	AC156435	708	0.89±0.07
TLR25	*Oreochromis niloticus*	ENSONIG00000014149	576	0.68±0.12

* Estimated TM-scores are used to measure the accuracy of structure modeling. A TM-score >0.5 indicates a model with the correct topology.

Supplementary Table S2. Module predictions for representative sequences of 28 types of vertebrate TLRs.

TLR*	1	1LA	1LB	2	2A	2B	3	4	5
**Signal**	1-24	1-27	1-29	1-20	1-25	1-22	1-22	1-23	1-20
**LRRNT**	25-46	28-49	30-76	21-53	26-64	23-53	23-52	24-55	21-47
**LRR1**	47-70	50-73	77-105	54-77	65-88	54-77	53-76	56-79	48-71
**LRR2**	71-94	74-97	106-132	78-101	89-112	78-101	77-100	80-103	72-96
**LRR3**	95-115	98-118	133-158	102-125	113-136	102-125	101-124	104-127	97-120
**LRR4**	116-140	119-143	159-187	126-150	137-161	126-150	125-148	128-151	121-146
**LRR5**	141-163	144-166	188-209	151-175	162-185	151-174	149-172	152-176	147-171
**LRR6**	164-188	167-189	210-233	176-199	186-209	175-198	173-198	177-204	172-197
**LRR7**	189-212	190-217	234-259	200-223	210-233	199-222	199-222	205-227	198-227
**LRR8**	213-245	218-244	260-284	224-250	234-260	223-249	223-249	228-254	228-254
**LRR9**	246-272	245-271	285-306	251-278	261-288	250-277	250-275	255-284	255-289
**LRR10**	273-298	272-310	307-329	279-308	289-317	278-305	276-299	285-309	290-313
**LRR11**	299-327	311-339	330-351	309-337	318-346	306-334	300-323	310-331	314-337
**LRR12**	328-349	340-361	352-375	338-361	347-370	335-358	324-356	332-352	338-361
**LRR13**	350-373	362-385		362-388	371-397	359-385	357-380	353-374	362-385
**LRR14**	374-399	386-411		389-414	398-423	386-411	381-408	375-400	386-404
**LRR15**	400-424	412-436		415-437	424-446	412-434	409-432	401-423	405-424
**LRR16**	425-446	437-458		438-458	447-467	435-455	433-457	424-448	425-449
**LRR17**	447-469	459-481		459-478	468-487	456-475	458-481	449-472	450-474
**LRR18**	470-491	482-503		479-500	488-509	476-497	482-507	473-497	475-503
**LRR19**	492-515	504-527		501-524	510-533	498-521	508-531	498-521	504-527
**LRR20**							532-563	522-545	528-549
**LRR21**							564-587	546-570	550-570
**LRR22**							588-611		
**LRR23**							612-636		
**LRR24**									
**LRR25**									
**LRRCT**	516-581	528-592	376-440	525-587	534-596	522-584	637-702	571-633	571-643
**TM**	582-604	593-615	441-463	588-610	597-619	585-607	703-725	634-656	644-666
**TIR**	605-786	616-818	464-652	611-784	620-793	608-781	726-904	657-839	667-858

Supplementary Table S2. Cont.

TLR*	5S	5SL	6	7	8	9	10	11	12
**Signal**	1-21	1-17	1-31	1-26	1-26	1-25	1-19	1-30	1-21
**LRRNT**	22-46	18-55	32-53	27-66	27-64	26-64	20-49	31-82	22-70
**LRR1**	47-70	56-79	54-77	67-90	65-88	65-88	50-73	83-105	71-93
**LRR2**	71-95	80-106	78-101	91-128	89-126	89-124	74-97	106-128	94-116
**LRR3**	96-119	107-130	102-122	129-149	127-147	125-144	98-118	129-156	117-143
**LRR4**	120-145	131-156	123-147	150-173	148-171	145-168	119-143	157-178	144-165
**LRR5**	146-170	157-181	148-170	174-205	172-202	169-200	144-166	179-218	166-200
**LRR6**	171-196	182-207	171-195	206-226	203-223	201-221	167-189	219-242	201-224
**LRR7**	197-228	208-234	196-219	227-250	224-247	222-245	190-213	243-265	225-247
**LRR8**	229-255	235-261	220-250	251-291	248-288	246-285	214-243	266-288	248-269
**LRR9**	256-289	262-297	251-277	292-315	289-312	286-309	244-270	289-313	270-293
**LRR10**	290-313	298-321	278-303	316-341	313-338	310-335	271-298	314-337	294-317
**LRR11**	314-338	322-345	304-332	342-371	339-368	336-365	299-327	338-362	318-343
**LRR12**	339-362	346-371	333-354	372-398	369-395	366-392	328-349	363-386	344-367
**LRR13**	363-386	372-395	355-378	399-422	396-419	393-416	350-373	387-410	368-391
**LRR14**	387-408	396-416	379-404	423-494	420-482	417-473	374-398	411-434	392-415
**LRR15**	409-431	417-439	405-429	495-518	483-506	474-497	399-422	435-458	416-439
**LRR16**	432-457	440-464	430-451	519-543	507-531	498-522	423-444	459-479	440-461
**LRR17**	458-484	465-491	452-474	544-567	532-555	523-546	445-467	480-503	462-486
**LRR18**	485-513	492-520	475-496	568-597	556-585	547-576	468-489	504-527	487-512
**LRR19**	514-537	521-544	497-520	598-620	586-609	577-599	490-513	528-555	513-541
**LRR20**	538-559	545-568		621-651	610-640	600-629		556-578	542-564
**LRR21**	560-580	569-592		652-676	641-665	630-654		579-607	565-593
**LRR22**				677-700	666-689	655-678		608-631	594-617
**LRR23**				701-724	690-713	679-702		632-656	618-642
**LRR24**				725-748	714-737	703-726			
**LRR25**				749-774	738-763	727-751			
**LRRCT**	581-641	593-652	521-586	775-842	764-825	752-818	514-576	657-718	643-709
**TM**			587-609	843-865	826-848	819-839	577-599	719-741	710-730
**TIR**			610-796	866-1049	849-1041	840-1032	600-811	742-926	731-906

Supplementary Table S2. Cont.

TLR*	13	14	15	18	19	20	21	22	23	25
**Signal**	1-68	1-25	1-22	1-22	1-31	1-18	1-38	1-22	1-21	1-27
**LRRNT**	69-104	26-56	23-55	23-53	32-79	19-64	39-74	23-59	22-57	28-63
**LRR1**	105-128	57-80	56-79	54-77	80-102	65-88	75-98	60-83	58-81	64-87
**LRR2**	129-152	81-104	80-103	78-101	103-126	89-111	99-122	84-107	82-105	88-111
**LRR3**	153-175	105-128	104-202	102-125	127-154	112-137	123-146	108-131	106-129	112-132
**LRR4**	176-199	129-153	203-233	126-150	155-178	138-161	147-170	132-155	130-153	133-157
**LRR5**	200-225	154-174	234-260	151-171	179-205	162-187	171-196	156-180	154-178	158-178
**LRR6**	226-248	175-198	261-288	172-195	206-234	188-219	197-219	181-205	179-204	179-208
**LRR7**	249-271	199-231	289-310	196-228	235-258	220-243	220-243	206-228	205-227	209-236
**LRR8**	272-295	232-255	311-334	229-252	259-281	244-263	244-267	229-255	228-250	237-259
**LRR9**	296-318	256-287	335-366	253-284	282-310	264-289	268-295	256-281	251-275	260-295
**LRR10**	319-348	288-315	367-387	285-311	311-335	290-313	296-318	282-306	276-301	296-321
**LRR11**	349-372	316-341	388-411	312-337	336-359	314-330	319-342	307-330	302-324	322-349
**LRR12**	373-397	342-365	412-431	338-361	360-385	331-372	343-370	331-354	325-348	350-376
**LRR13**	398-421	366-394	432-454	362-390	386-409	373-396	371-395	355-377	349-371	377-402
**LRR14**	422-445	395-420	455-476	391-416	410-433	397-420	396-419	378-401	372-395	403-428
**LRR15**	446-469	421-444	477-498	417-440	434-457	421-444	420-443	402-425	396-419	429-450
**LRR16**	470-493	445-467	499-520	441-462	458-481	445-468	444-467	426-449	420-443	451-472
**LRR17**	494-517	468-491	521-544	463-486	482-508	469-496	468-491	450-473	444-467	473-496
**LRR18**	518-541	492-513	545-566	487-508	509-532	497-521	492-515	474-497	468-491	497-518
**LRR19**	542-565	514-537	567-590	509-532	533-555	522-544	516-540	498-525	492-512	519-542
**LRR20**	566-594				556-579	545-568	541-569	526-550	513-537	
**LRR21**	595-620				580-601	569-590	570-596	551-576	538-566	
**LRR22**	621-644				602-630	591-619	597-620	577-603	567-593	
**LRR23**	645-672				631-654	620-643	621-646	604-630	594-617	
**LRR24**	673-696				655-678	644-667	647-670	631-654	618-641	
**LRR25**	697-720						671-694	655-678	642-665	
**LRRCT**	721-783	538-600	591-653	533-598	679-745	668-733	695-752	679-742	666-729	543-603
**TM**	784-804	601-622	654-676	599-621	746-763	734-753	753-775	743-765	730-753	604-626
**TIR**	805-991	623-836	677-868	622-854	764-955	754-933	776-972	766-947	754-941	627-827

* See Supplementary Table S1 for information regarding the sequences used for module predictions. “Signal” represents the signal peptide. “TM” represents the transmembrane region. “TIR” represents the whole intracellular domain.

Supplementary Table S3. Typical ligands of vertebrate TLRs.

TLR	Family	Localization	Dimerization	Ligand	Origin of ligand	Ref.
TLR1/TLR2	TD family 1	Cell surface	Heterodimer	Lipopeptides, lipomannan	Bacteria	(1-4)
TLR6/TLR2	TD family 1	Cell surface	Heterodimer	Lipopeptides	Bacteria, mycoplasma	(5-7)
TLR10/TLR2	TD family 1	Cell surface	Heterodimer	Lipopeptides	Bacteria	(8)
TLR1LA/TLR2A, TLR1LA/TLR2B, TLR1LB/TLR2A, TLR1LB/TLR2B	TD family 1	Cell surface	Heterodimer	Lipopeptides, peptidoglycan	Bacteria, mycoplasma	(9, 10)
TLR4	TD family 1	Cell surface	Homodimer	LPS	Bacteria	(11, 12)
TLR3	SD family 3	Endosome	Homodimer	dsRNA	Virus	(13)
TLR5	SD family 5	Cell surface	Homodimer	Flagellin	Flagellated bacteria	(14-18)
TLR5S	SD family 5	Soluble		Flagellin	Flagellated bacteria	(19, 20)
TLR7	SD family 7	Endosome	Homodimer	ssRNA	Virus	(21-23)
TLR8	SD family 7	Endosome	Homodimer	ssRNA	Virus	(23)
TLR9	SD family 7	Endosome	Homodimer	CpG DNA	Virus, bacteria	(24-26)
TLR13	SD family 13	Endosome		ssRNA	Virus, bacteria	(27-30)
TLR21	SD family 13	Endosome		CpG DNA	Virus, bacteria	(26, 31)
TLR22	SD family 13	Cell surface		dsRNA	Virus	(32)
TLR15	SD family 15	Cell surface		Proteases	Bacteria, fungi	(33)
TLR11/TLR12, TLR11, TLR12	TTD family 11	Endosome	Homodimer or heterodimer	Profilin, flagellin	*Toxoplasma gondii, Salmonella Typhi*	(34-37)
TLR19, TLR20	TTD family 11	Intracellular compartment		unknown	Protozoan parasite	(38, 39)

References

1. Aliprantis AO, *et al.* (1999) Cell activation and apoptosis by bacterial lipoproteins through toll-like receptor-2. *Science* 285(5428):736-739.
2. Takeuchi O, *et al.* (2002) Cutting edge: role of Toll-like receptor 1 in mediating immune response to microbial lipoproteins. *Journal of immunology* 169(1):10-14.
3. Quesniaux VJ, *et al.* (2004) Toll-like receptor 2 (TLR2)-dependent-positive and TLR2-independent-negative regulation of proinflammatory cytokines by mycobacterial lipomannans. *Journal of immunology* 172(7):4425-4434.
4. Vignal C, *et al.* (2003) Lipomannans, but not lipoarabinomannans, purified from Mycobacterium chelonae and Mycobacterium kansasii induce TNF-alpha and IL-8 secretion by a CD14-toll-like receptor 2-dependent mechanism. *Journal of immunology* 171(4):2014-2023.
5. Takeuchi O, *et al.* (2001) Discrimination of bacterial lipoproteins by Toll-like receptor 6. *International immunology* 13(7):933-940.
6. Bulut Y, Faure E, Thomas L, Equils O, & Arditi M (2001) Cooperation of Toll-like receptor 2 and 6 for cellular activation by soluble tuberculosis factor and Borrelia burgdorferi outer surface protein A lipoprotein: role of Toll-interacting protein and IL-1 receptor signaling molecules in Toll-like receptor 2 signaling. *Journal of immunology* 167(2):987-994.
7. Buwitt-Beckmann U, *et al.* (2005) Toll-like receptor 6-independent signaling by diacylated lipopeptides. *European journal of immunology* 35(1):282-289.
8. Guan Y, *et al.* (2010) Human TLRs 10 and 1 share common mechanisms of innate immune sensing but not signaling. *Journal of immunology* 184(9):5094-5103.
9. Higuchi M, *et al.* (2008) Combinational recognition of bacterial lipoproteins and peptidoglycan by chicken Toll-like receptor 2 subfamily. *Developmental and comparative immunology* 32(2):147-155.
10. Keestra AM, de Zoete MR, van Aubel RA, & van Putten JP (2007) The central leucine-rich repeat region of chicken TLR16 dictates unique ligand specificity and species-specific interaction with TLR2. *Journal of immunology* 178(11):7110-7119.
11. Shimazu R, *et al.* (1999) MD-2, a molecule that confers lipopolysaccharide responsiveness on Toll-like receptor 4. *The Journal of experimental medicine* 189(11):1777-1782.
12. Poltorak A, *et al.* (1998) Defective LPS signaling in C3H/HeJ and C57BL/10ScCr mice: mutations in Tlr4 gene. *Science* 282(5396):2085-2088.
13. Alexopoulou L, Holt AC, Medzhitov R, & Flavell RA (2001) Recognition of double-stranded RNA and activation of NF-kappaB by Toll-like receptor 3. *Nature* 413(6857):732-738.
14. Hayashi F, *et al.* (2001) The innate immune response to bacterial flagellin is mediated by Toll-like receptor 5. *Nature* 410(6832):1099-1103.
15. Eaves-Pyles TD, Wong HR, Odoms K, & Pyles RB (2001) Salmonella flagellin-dependent proinflammatory responses are localized to the conserved amino and carboxyl regions of the protein. *Journal of immunology* 167(12):7009-7016.
16. Mizel SB, West AP, & Hantgan RR (2003) Identification of a sequence in human toll-like receptor 5 required for the binding of Gram-negative flagellin. *The Journal of biological chemistry* 278(26):23624-23629.
17. Iqbal M, *et al.* (2005) Identification and functional characterization of chicken toll-like receptor 5 reveals a fundamental role in the biology of infection with Salmonella enterica serovar typhimurium. *Infection and immunity* 73(4):2344-2350.
18. Stockhammer OW, Zakrzewska A, Hegedus Z, Spaink HP, & Meijer AH (2009) Transcriptome profiling and functional analyses of the zebrafish embryonic innate immune response to Salmonella infection. *Journal of immunology* 182(9):5641-5653.
19. Tsujita T, *et al.* (2004) Sensing bacterial flagellin by membrane and soluble orthologs of Toll-like receptor 5 in rainbow trout (Onchorhynchus mikiss). *The Journal of biological chemistry* 279(47):48588-48597.
20. Munoz I, Sepulcre MP, Meseguer J, & Mulero V (2013) Molecular cloning, phylogenetic analysis and functional characterization of soluble Toll-like receptor 5 in gilthead seabream, Sparus aurata. *Fish & shellfish immunology* 35(1):36-45.
21. Lund JM, *et al.* (2004) Recognition of single-stranded RNA viruses by Toll-like receptor 7. *Proceedings of the National*

Academy of Sciences of the United States of America 101(15):5598-5603.

22. Diebold SS, Kaisho T, Hemmi H, Akira S, & Reis e Sousa C (2004) Innate antiviral responses by means of TLR7-mediated recognition of single-stranded RNA. *Science* 303(5663):1529-1531.
23. Heil F*, et al.* (2004) Species-specific recognition of single-stranded RNA via toll-like receptor 7 and 8. *Science* 303(5663):1526-1529.
24. Rutz M*, et al.* (2004) Toll-like receptor 9 binds single-stranded CpG-DNA in a sequence- and pH-dependent manner. *European journal of immunology* 34(9):2541-2550.
25. Hemmi H*, et al.* (2000) A Toll-like receptor recognizes bacterial DNA. *Nature* 408(6813):740-745.
26. Yeh DW*, et al.* (2013) Toll-like receptor 9 and 21 have different ligand recognition profiles and cooperatively mediate activity of CpG-oligodeoxynucleotides in zebrafish. *Proceedings of the National Academy of Sciences of the United States of America* 110(51):20711-20716.
27. Li XD & Chen ZJ (2012) Sequence specific detection of bacterial 23S ribosomal RNA by TLR13. *eLife* 1:e00102.
28. Shi Z*, et al.* (2011) A novel Toll-like receptor that recognizes vesicular stomatitis virus. *The Journal of biological chemistry* 286(6):4517-4524.
29. Oldenburg M*, et al.* (2012) TLR13 recognizes bacterial 23S rRNA devoid of erythromycin resistance-forming modification. *Science* 337(6098):1111-1115.
30. Hidmark A, von Saint Paul A, & Dalpke AH (2012) Cutting edge: TLR13 is a receptor for bacterial RNA. *Journal of immunology* 189(6):2717-2721.
31. Keestra AM, de Zoete MR, Bouwman LI, & van Putten JP (2010) Chicken TLR21 is an innate CpG DNA receptor distinct from mammalian TLR9. *Journal of immunology* 185(1):460-467.
32. Matsuo A*, et al.* (2008) Teleost TLR22 recognizes RNA duplex to induce IFN and protect cells from birnaviruses. *Journal of immunology* 181(5):3474-3485.
33. de Zoete MR, Bouwman LI, Keestra AM, & van Putten JP (2011) Cleavage and activation of a Toll-like receptor by microbial proteases. *Proceedings of the National Academy of Sciences of the United States of America* 108(12):4968-4973.
34. Yarovinsky F*, et al.* (2005) TLR11 activation of dendritic cells by a protozoan profilin-like protein. *Science* 308(5728):1626-1629.
35. Zhang D*, et al.* (2004) A toll-like receptor that prevents infection by uropathogenic bacteria. *Science* 303(5663):1522-1526.
36. Mathur R*, et al.* (2012) A mouse model of Salmonella typhi infection. *Cell* 151(3):590-602.
37. Koblansky AA*, et al.* (2013) Recognition of profilin by Toll-like receptor 12 is critical for host resistance to Toxoplasma gondii. *Immunity* 38(1):119-130.
38. Pietretti D*, et al.* (2014) Identification and functional characterization of nonmammalian Toll-like receptor 20. *Immunogenetics* 66(2):123-141.
39. Lee PT*, et al.* (2014) Identification and characterisation of TLR18-21 genes in Atlantic salmon (Salmo salar). *Fish & shellfish immunology* 41(2):549-559.

Supplementary Table S4. Distribution of TLR genes in 39 vertebrate genomes.

No.	Common name	Scientific name	Taxon	Ensembl assembly	TLR gene copies*		
					TD	SD	TTD
1	Human	*Homo sapiens*	Mammalia	GRCh38.p2	5	5	0
2	Chimpanzee	*Pan troglodytes*	Mammalia	CHIMP2.1.4	5	4	0
3	Gorilla	*Gorilla gorilla gorilla*	Mammalia	gorGor3.1	6	5	0
4	Gibbon	*Nomascus leucogenys*	Mammalia	Nleu1.0	4	5	0
5	Macaque	*Macaca mulatta*	Mammalia	MMUL 1.0	5	5	0
6	Marmoset	*Callithrix jacchus*	Mammalia	C_jacchus3.2.1	5	5	0
7	Bushbaby	*Otolemur garnettii*	Mammalia	OtoGar3	4	5	0
8	Mouse	*Mus musculus*	Mammalia	GRCm38.p3	4	6	2
9	Rat	*Rattus norvegicus*	Mammalia	Rnor_5.0	5	5	2
10	Squirrel	*Ictidomys tridecemlineatus*	Mammalia	spetri2	5	6	1
11	Guinea Pig	*Cavia porcellus*	Mammalia	cavPor3	5	4	0
12	Cow	*Bos taurus*	Mammalia	UMD3.1	5	4	0
13	Sheep	*Ovis aries*	Mammalia	Oar_v3.1	5	5	0
14	Pig	*Sus scrofa*	Mammalia	Sscrofa10.2	5	5	0
15	Horse	*Equus caballus*	Mammalia	Equ Cab 2	5	4	2
16	Microbat	*Myotis lucifugus*	Mammalia	Myoluc2.0	4	6	1
17	Dog	*Canis lupus familiaris*	Mammalia	CanFam3.1	5	5	0
18	Ferret	*Mustela putorius furo*	Mammalia	MusPutFur1.0	4	5	0
19	Panda	*Ailuropoda melanoleuca*	Mammalia	ailMel1	5	5	0
20	Elephant	*Loxodonta africana*	Mammalia	Loxafr3.0	5	5	2
21	Armadillo	*Dasypus novemcinctus*	Mammalia	Dasnov3.0	5	4	2
22	Tasmanian devil	*Sarcophilus harrisii*	Mammalia	Devil_ref v7.0	4	6	0
23	Platypus	*Ornithorhynchus anatinus*	Mammalia	OANA5	4	4	1
24	Chicken	*Gallus gallus*	Aves	Galgal4	5	5	0
25	Turkey	*Meleagris gallopavo*	Aves	Turkey_2.01	5	5	0
26	Duck	*Anas platyrhynchos*	Aves	BGI_duck_1.0	5	4	0
27	Zebra Finch	*Taeniopygia guttata*	Aves	taeGut3.2.4	5	4	0
28	Flycatcher	*Ficedula albicollis*	Aves	FicAlb_1.4	5	5	0
29	Anole lizard	*Anolis carolinensis*	Reptiles	AnoCar2.0	6	7	0
30	Chinese softshell turtle	*Pelodiscus sinensis*	Reptiles	PelSin_1.0	4	10	0
31	Xenopus	*Xenopus tropicalis*	Amphibia	JGI 4.2	9	10	1
32	Coelacanth	*Latimeria chalumnae*	Coelacanthimorpha	LatCha1	4	11	0
33	Spotted gar	*Lepisosteus oculatus*	Actinopterygii	LepOcu1	6	8	0
34	Zebrafish	*Danio rerio*	Actinopterygii	Zv9	6	10	4
35	Cave fish	*Astyanax mexicanus*	Actinopterygii	AstMex102	3	11	3
36	Fugu	*Takifugu rubripes*	Actinopterygii	FUGU 4.0	3	9	0
37	Medaka	*Oryzias latipes*	Actinopterygii	HdrR	4	7	0
38	Platyfish	*Xiphophorus maculatus*	Actinopterygii	Xipmac4.4.2	3	13	0
39	Tilapia	*Oreochromis niloticus*	Actinopterygii	Orenil1.0	3	9	0

* Three-domain TLRs (TD), single-domain TLRs (SD), and trans-three-domain TLRs (TTD).

Supplementary Data S1. Information for 1428 TLRs in vertebrates. Columns: (A) The accession numbers of the coding sequences for TLRs; (B) Scientific names of species from which the TLRs originated; (C) The taxonomic designations of species from which the TLRs originated; (D) TLR types; (E) TLR families; (F) The architectures of TLR ectodomains; (G) The coding sequences of the TLRs.
